# Sb–TiO_2_/C nanofiber paper as a flexible high-performance anode for lithium-ion and sodium-ion batteries

**DOI:** 10.1039/d6ra02199a

**Published:** 2026-05-05

**Authors:** Kun Su, Jinhua Dai

**Affiliations:** a School of Pharmacy, Baotou Medical College 010040 Baotou Inner Mongolia China; b School of Information Science and Technology, Baotou Teachers' College 012000 Baotou Inner Mongolia China djh19@126.com

## Abstract

Titanium dioxide (TiO_2_) is regarded as a promising anode material for lithium-ion batteries (LIBs) and sodium-ion batteries (SIBs) due to its low cost, non-toxicity, high safety, and abundant reserves. However, its practical application is severely limited by poor electrical conductivity, low reversible capacity, and sluggish ion diffusion kinetics. To address these critical issues, we successfully synthesized Sb–TiO_2_/C nanofiber paper *via* electrospinning combined with a subsequent calcination, with the core design feature of constructing a rutile/anatase heterojunction that significantly enhances ion and electron transfer kinetics and provides abundant active sites for Li^+^/Na^+^ storage. When used as a freestanding anode without any binders, conductive additives, or current collectors, it delivers a high and stable specific capacity of ∼291 mA h g^−1^ at a current density of 500 mA g^−1^ after 500 cycles for LIBs and a stable specific capacity of 162 mA h g^−1^ at 500 mA g^−1^ after 300 cycles for SIBs, which highlights the critical role of the rutile/anatase heterojunction in optimizing the electrochemical performance of TiO_2_-based anodes, establishing the prepared Sb–TiO_2_/C nanofiber paper as a promising candidate for flexible LIBs and SIBs. This work provides a feasible and scalable strategy for the practical application of TiO_2_-based materials in next-generation flexible energy storage devices.

## Abstract 2

Flexible, freestanding Sb–TiO_2_/C nanofiber paper featuring an *in situ* constructed rutile/anatase heterojunction serves as a high-performance anode for both lithium- and sodium-ion batteries.

## Graphical abstract

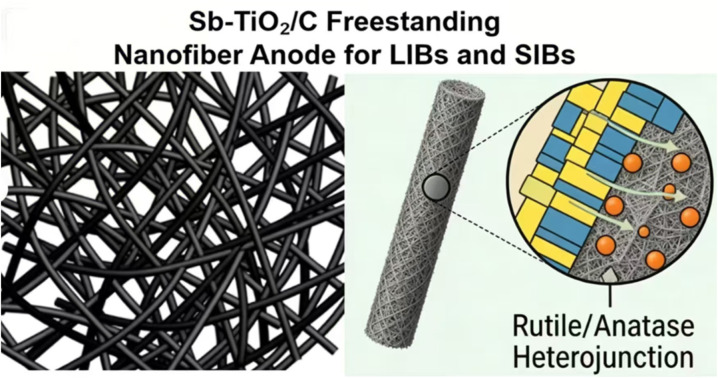

## 1. Introduction

Nowadays, more and more flexible electronic devices have come into our daily lives, such as flexible mobile phones, flexible televisions and wearable devices. All these applications rely on the support of batteries. Therefore, flexible electrodes have become an inevitable choice for flexible electronic devices, and their performance directly determines the service life and application effect of flexible electronic devices.^1,2^ An ideal flexible electrode should possess a long cycling life, be able to attach to human skin and transmit detection signals in real-time,^3–5^ and maintain structural integrity after repeated bending and unfolding, which can greatly improve the overall performance of batteries in practical applications. Although various flexible electrodes have been successfully developed,^6–10^ they still suffer from several drawbacks. For instance, they tend to fracture during repeated bending, leading to the detachment of active materials. This not only accelerates capacity fading but may also cause short circuits by piercing the separator. Thus, it is imperative to develop electrode materials with excellent electrochemical performance and good flexibility for practical batteries.

TiO_2_-based nano materials (including anatase, rutile, bronze phase, Hollandite-type and other crystal forms), have, attracted extensive attention and been used as anodes for LIBs and SIBs due to their stable structure, environmental friendliness and high safety.^11–14^ However, the above forms of titanium dioxide exhibit low specific capacity (below 200 mA h g^−1^), poor electrical conductivity (∼10^−12^ S cm^−1^), low ion diffusion coefficient and inferior flexibility,^15^ which greatly hinder their application in flexible batteries.

At present, many effective strategies could be used to solve the above problems of TiO_2_-based flexible anodes. Constructing heterostructures, doping elements and compositing carbon matrix are the main effective modification methods.^4^ Meanwhile, fabricating binder-free self-supporting nanofiber structures can further optimize the mechanical flexibility and electrochemical performance of TiO_2_-based electrodes, which has been verified in previous reports.^16^ Heterointerface engineering has been proven to effectively improve the electrochemical performance of electrode materials. Benefiting from strong interfacial chemical bonding, carbon hybridization and electrospun nanofiber structures, flexible electrodes show enhanced charge transfer kinetics and structural stability.^16–19^ In addition, the “self-protection” characteristic of heterostructured electrodes can further improve the structural stability of electrode materials.^20–22^ The combination of heterointerface engineering and defect regulation strategies can effectively enhance the electrochemical performance of alkali metal-ion batteries.^23–25^ Strategies such as constructing abundant heterojunctions, introducing anion vacancies and designing carbon-based hybrid structures have been verified to effectively optimize charge transport pathways and strengthen structural stability.^23–25^ Another effective strategy is to introduce metallic elements (such as Sb). Antimony (Sb), as a typical alloy-type anode with high theoretical capacity, also faces two core bottlenecks in practical application: the huge volume change (∼200%) during repeated alloying/dealloying processes easily leads to the pulverization of active materials and rapid capacity decay;^26^ meanwhile, continuous volume fluctuation causes repeated rupture and re-growth of the solid electrolyte interphase (SEI) film, resulting in low initial coulombic efficiency and poor cycling stability.^27^ These challenges are also key factors restricting the commercial application of alloy-type flexible anodes.^28^ Electrospinning is another facile and scalable strategy to prepare flexible self-supporting nanofiber electrodes. This method can realize carbon matrix composite while constructing self-standing nanofiber structures.^17^ The construction of hierarchical porous nanostructures and flexible self-standing architectures can further shorten ion diffusion paths and accommodate structural stress during cycling.^25,29^ At last, other strategies such as heteroatom doping, introducing high-entropy materials^30^ and oxygen vacancy engineering have been demonstrated as effective approaches to tailor the electronic structure, enrich active sites and accelerate surface pseudocapacitive kinetics of metal oxide anodes.^31^

However, although TiO_2_-based nanofibers prepared by electrospinning have attracted wide attention owing to their high safety and structural stability, their poor intrinsic conductivity and slow ion diffusion kinetics still limit their practical application.^31^ Herein, we propose a synergistic modification strategy: introducing antimony (Sb) as an alloy-type component to boost the capacity and redox reactivity of TiO_2_, and *in situ* constructing rutile/anatase heterojunctions to optimize interfacial charge transfer. Benefiting from the above compositional and structural design, the as-prepared flexible self-supporting Sb–TiO_2_/C electrode is expected to achieve high capacity, fast reaction kinetics and excellent structural stability for both lithium-ion and sodium-ion storage.

## 2. Experimental section

### 2.1 Fabrication of Sb–TiO_2_/C nanofibers paper

**Fig. 1:**
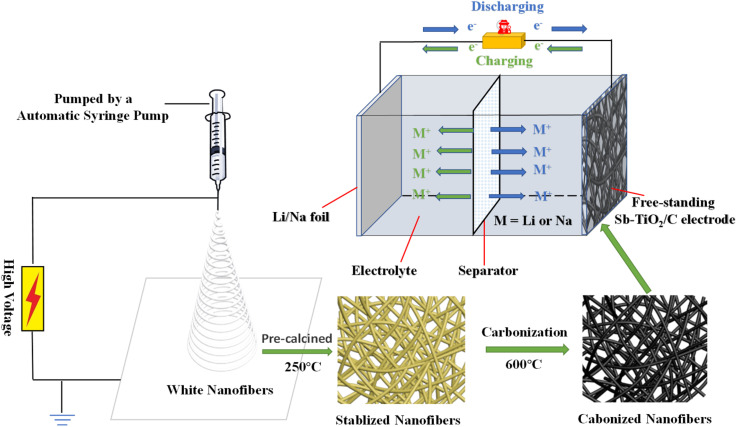
The synthesis process of the Sb–TiO_2_/C nanofibers paper.

All the composite nanofibers were made through electrospinning followed by a post-calcination process (see Fig. 1). To fabricate Sb–TiO_2_/C nanofibers (NFs) paper, a precursor solution for electrospinning was made before the electrospinning. Initially, 9 mL *N*,*N*-dimethylformamide (DMF), and 1 mL titanium(iv) isopropoxide (TTIP, C_12_H_28_O_4_Ti) were mixed homogeneously. Then 0.2 g antimony(iii) trichloride dihydrate (SbCl_3_) was added into the previously mentioned solution under stirring, and then 0.7 g polyvinylpyrrolidone (PVP, *M*_w_ = 1 300 000) was added into the solution, magnetic stirring for 10 h until a transparent precursor solution was formed. The precursor solution was inhaled into a 10 mL syringe with a blunt stainless-steel needle and spun on the homemade electrospinning unit. The applied voltage was 15 kV; the needle-to-collector distance was maintained at 12 cm. The flow rate was 0.3 mL h^−1^. After the electrospinning process, it was collected, dried at 70 °C for 8 h under vacuum, and pre-calcined at 250 °C for 2 h with a heating rate of 2 °C min^−1^. Finally, the Sb–TiO_2_/C NFs paper can be obtained by carbonization at 600 °C for 4 h in Argon atmosphere with a heating rate of 3 °C min^−1^. Identical processes were utilized to prepare the other Sb–TiO_2_/C NFs paper and the pristine TiO_2_/C NFs paper except for the different amounts of SbCl_3_ added (0 g SbCl_3_for the pristine NFs, 0.2 g, and 0.4 g for two kinds of Sb–TiO_2_/C NFs, respectively).

### 2.2 Material characterizations

The phases and crystallinity of the as-made samples were confirmed by X-ray powder diffraction (XRD) executed on a Panalytical Empyrean diffractometer with Cu-Kα radiation (*λ* = 1.5418 Å). The morphologies and microstructures were identified by using a scanning electron microscope (SEM, NanoSEM, 4300). The images of the transmission electron microscope (TEM), high-resolution transmission electron microscope (HRTEM), and High-Angle Annular Dark Field (HAADF) were recorded on JEOL 2100f (operating voltage: 200 kV). X-ray photoelectron spectroscopy (XPS, Thermo, ESCALAB 250XI) was utilized to examine the elemental states of all the samples in this work. Thermogravimetric Analysis (TGA) was executed through a Thermal Gravimetric Analyzer (NETZSCH TG209F1 Libra). Nitrogen adsorption–desorption isotherms were measured on Quantachrome Autosorb Station 2. Inductively Coupled Plasma Optical Emission Spectrometer (ICP-OES/MS PerkinElmer 8300) was used to determine the component of titanium and antimony in composite nanofibers.

### 2.3 Electrochemical measurements

As a kind of freestanding and conductive material, Sb–TiO_2_/C nanofibers paper was directly employed to assemble half-cells for electrochemical performance assessment. All over the processes, none of the conductive additives, binders, and current collectors were used. CR2025 coin cells were utilized to fabricate the half-cell with lithium or sodium metal as the counter/reference electrode and the Sb–TiO_2_/C nanofibers paper, which were punched into disks with a diameter of 1 cm, as the working electrodes. Different electrolytes and separators were used for LIBs and SIBs. 1 mol L^−1^ LiPF_6_ in a mixture of dimethyl carbonate (DMC) and ethylene carbonate (EC) with 1 : 1 volumetric ratio was employed as electrolyte and the Celgard 2300 microporous paper as a separator for lithium-ion half-cell. 1 mol L^−1^ NaClO_4_ in a mixture of ethylene carbonate (EC)/propylene carbonate (PC) (1 : 1 vol%) with 5% fluorinated ethylene carbonate (FEC, additives) was uase as electrolyte and glass fiber films (Whatman GF/D) were used as separators for Sodium-ion half-cells. All the coin cells were assembled in a glove box with argon filled and both water and Oxygen contents kept below 0.2 ppm. Galvanostatic discharge/charge tests both in lithium-ion half-cells and sodium-ion half-cells were executed on the CT2001A battery testing system (LAND, China). Cyclic voltammetry tests (CV) were conducted on a CHI760E electrochemical workstation (Chenhua, Shanghai, China) at a scan rate of 0.1 mV s^−1^.

The specific capacity of the electrode was calculated based on the total mass of the self-standing nanofiber paper disk (diameter: 1 cm, average mass loading: 1.2–1.5 mg cm^−2^), which is the standard method for freestanding electrodes. The mass of each electrode disk was accurately weighed with a high-precision electronic balance (precision: 0.01 mg) before cell assembly, to ensure the accuracy and comparability of the specific capacity data.

## 3. Results and discussion

### 3.1 Morphology and structural characterization

**Fig. 2:**
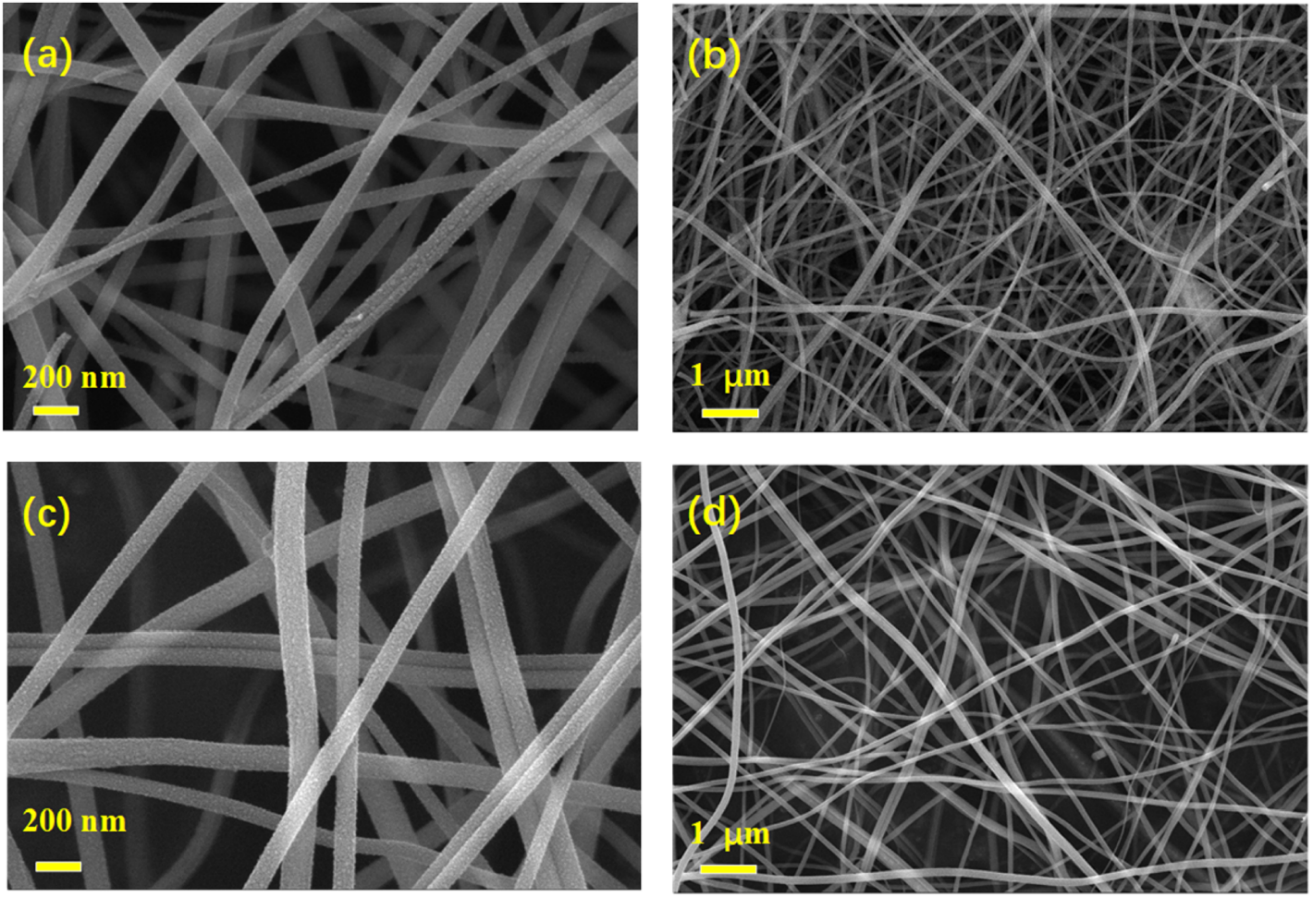
SEM images of TiO_2_/C NFs (a and c) and the Sb–TiO_2_/C NFs (b and d).

The incorporation of metal or non-metal elements can enhance the performance of TiO_2_ anodes antimony (Sb) is one kind of metal that is attracting extensive attention. It has a high theoretical capacity (660 mA h g^−1^ or 1890 mA h cm^−3^), unique puckered-layer structures, small electrode polarization (≈0.2 V), and moderate working voltage (0.8–0.9 V).^32^ All the characteristics indicate that Sb is one of the most promising metal as a flexible anode material. Because of this, Sb metal was selected as an additive metal to improve the performance of TiO_2._Fig. 1 elucidates the fabrication process of porous Sb–TiO_2_/C nanofibers paper. Initially, specific amounts of SbCl_3_, DMF, TTIP, and PVP were mixed homogeneously to produce the precursor solution, which was electrospun into a white nanofibers paper (Fig. S1a, SI). Subsequently, the as-spun white fibers paper was heated to 250 °C kept for 2 h in the air to stabilize the nanofibers' structure, resulting in light brown nanofibers paper (Fig. S1b). Eventually, the stabilized paper was loaded into a tube furnace and heated under Argon at 600 °C to gain the porous Sb–TiO_2_/C nanofibers paper (Fig. S1c). Scanning electron microscopy (SEM) was utilized to visualize the morphology of Sb–TiO_2_/C and TiO_2_/C nanofibers, as is shown in Fig. 2a–d. Little aggregated particles can be observed on the surface of the nanofibers, which implies TiO_2_ and Sb species are homogeneously embedded in the carbon matrix, which is corresponding with the XRD results. Both TiO_2_/C and Sb–TiO_2_/C (0.2 g SbCl_3_) samples showed a similar morphology of nanofibers and lengths up to several millimeters with 100–200 nm diameter. It is worth noting that when the amount of SbCl_3_ added was increased to 0.4 g, the products showed plenty of aggregation plots (Fig. S2). To evaluate the flexibility of the as-fabricated Sb–TiO_2_/C nanofiber paper, (Fig. S3), the porous Sb–TiO_2_/C nanofiber paper could almost completely intact after repeated folding in half multiple times, demonstrating its outstanding mechanical flexibility and structural stability. Conversely, Sb–TiO_2_/C (0.4 g SbCl_3_) nanofibers showed poor flexibility compared to the Sb–TiO_2_/C (0.2 g SbCl_3_) nanofibers. Good flexibility of electrodes for battery applications is one of the most needed prerequisites. Therefore, 0.2 g of SbCl_3_ is the optimal option to prepare nanofibers in this work.

**Fig. 3:**
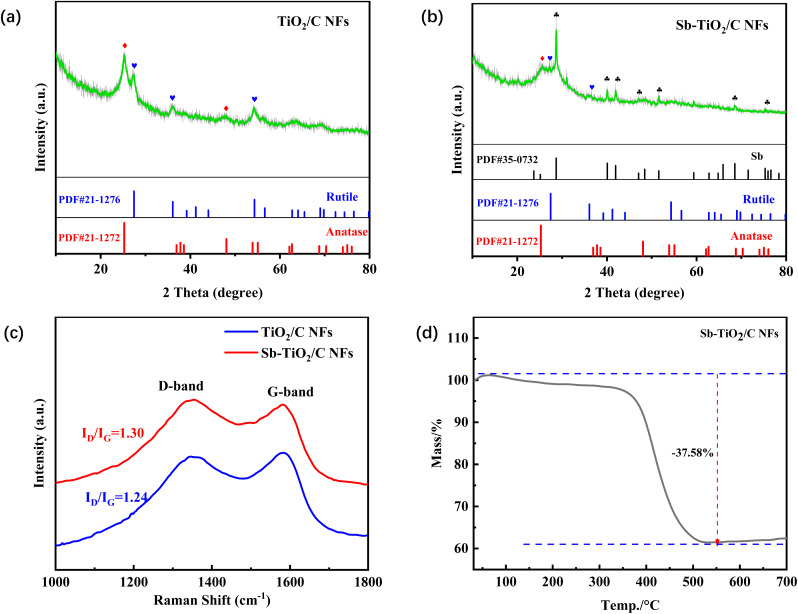
XRD patterns (a and b), Raman spectrums (c), and TGA-curve of TiO_2_/C NFs and Sb–TiO_2_/C NFs (d).

Powder X-ray diffraction (XRD) pattern is collected to analyze the phases of as-made nanofibers, as is shown in Fig. 3a and b. XRD analysis reveals the presence of two TiO_2_ phases both in the TiO_2_/C NFs and Sb–TiO_2_/C NFs samples. One is the anatase phase (JCPDS card no. 21-1272) and the other is the rutile phase (JCPDS card no. 21-1276). In the Sb–TiO_2_/C NFs sample, the peaks at 28.6°, 40.1°, 41.9°, and 51.7° (Fig. 3b) can be indexed to metallic antimony (JCPDS card no. 35-0732), the peak at 27.3° (110), 36.0° (101) refers to rutile phase, and the peak at 25.3° is assigned to the (101) plane of anatase phase, respectively. XRD patterns indicate no relative peaks assigned to graphene and other carbon species. This is reasonable probably due to its amorphous morphology. The Raman spectra of two kinds of NFs (Fig. 3c) show two strong peaks at about 1350 and 1580 cm^−1^, corresponding to a disorder-induced feature (D-band) and the E_2g_ mode of graphite (G-band), respectively.^33,34^ As shown on the fitting curve (Fig. S4), the values of *I*_D_/*I*_G_ for TiO_2_/C NFs and Sb–TiO_2_/C NFs are 1.24 and 1.30 respectively, which indicates a higher degree of defects in the carbon, potentially providing more intercalation site for Li-storage.^35^ The fingerprint region of Raman spectra of these two samples is shown in Fig. S5. For both samples, the characteristic peaks located at ∼144, 395, 515, and 639 cm^−1^ are assigned to the E_g_, B_1g_, A_1g_, and E_g_ vibrational modes of anatase TiO_2_, respectively, while the peaks at ∼447 and 612 cm^−1^ correspond to the E_g_ and A_1g_ modes of rutile TiO_2_, confirming the coexistence of rutile and anatase phases in the Sb–TiO_2_/C composite. The peaks located at ∼1350 and 1580 cm^−1^ are the D band and G band of carbon, respectively, indicating the successful carbonization of the nanofiber matrix. Notably, no independent characteristic peaks of metallic Sb or Sb–O bonds are observed in the fingerprint region below 1000 cm^−1^ for the Sb–TiO_2_/C sample, which is attributed to the low Sb content (7.64 wt% quantified by ICP-OES) and the strong masking effect of the TiO_2_ characteristic peaks. To further confirm the existence form and bonding environment of Sb, joint analysis of XRD, XPS, and ICP-OES was carried out. The XRD pattern shows the characteristic diffraction peaks of metallic Sb (JCPDS no. 35-0732), and the high-resolution Sb 3d XPS spectrum confirms the existence of Sb^3+^ species, indicating that Sb mainly exists as microcrystalline metallic Sb embedded in the TiO_2_/C matrix, with a small amount of Sb bonded to TiO_2_ in the form of Sb–O bonds. The ICP-OES result accurately quantifies the Sb content, which is consistent with the above characterization and explains the masking of Sb-related Raman signals. Gravimetric analysis (TGA) is carried out in the air to determine the chemical composition in the samples. As shown on the TG cure (Fig. 3d), a major weight loss appears between 350–550 °C which may refer to the oxidation of carbon in the matrix. The total weight loss is approximately 37.5 wt%, so the total Sb–TiO_2_ weight ratio can be estimated to 62.5 wt%. The element composition of Sb–TiO_2_/C was further determined by an inductively coupled plasma optical emission spectrometer (ICP-OES). The weight ratio of Sb and Ti measured by ICP-OES is 7.64%, 30.78%, which comply with the results of TGA.

### 3.2 Pore structure and surface chemical analysis

Pore width and pore size distribution are measured by N_2_ adsorption–desorption experiments. As shown in the relative isotherms (insets in Fig. S6), the BET surface area of Sb–TiO_2_/C nanofibers is estimated to be 153.8 m^2^ g^−1^ by the methodology of multi-point BET, which is larger than that of TiO_2_/C nanofibers (143.6 m^2^ g^−1^). From the pore size distribution curves (BJH adsorption) in Fig. S6, Sb–TiO_2_/C nanofibers have an obvious pore-size peak at 3.315 nm, which is smaller than that of TiO_2_/C nanofibers(3.771 nm). The pore volume of Sb–TiO_2_/C samples and TiO_2_/C ones are approximately the same (0.097 cm^−3^ g^−1^). All the results acquired indicate that plenty of micropores disperse inside both the Sb–TiO_2_/C and TiO_2_/C nanofibers. That is, the nanofibers have a porous structure with 3d intercrossed networks, which may mitigate the volume expansion effect caused by Sb–Li alloying reactions, and the large BET surface area of porous Sb–TiO_2_/C nanofibers is beneficial to supplying enough contact areas for the electrode and the electrolyte. Compared to the antimony-undoped samples, Sb–TiO_2_/C ones with larger BET surface area and smaller pore width are expected to show better electrochemical performance. Also, when the addition amount of Sb increased to 0.4 g, the BET surface area decreased dramatically (see Fig. S6c), about 74.8 m^2^ g^−1^, which is probably due to its blocky morphology (Fig. S2). The evolution of pore structure can be well interpreted by the regulating effect of Sb content. Moderate Sb doping effectively suppresses the grain growth of TiO_2_ nanocrystals, which creates abundant mesopores and micropores, thus increasing the specific surface area and reducing the average pore size. However, excessive Sb loading causes severe particle aggregation and pore blockage, leading to an obvious decrease in specific surface area. Such a well-tailored porous structure not only shortens ion diffusion pathways and provides sufficient active sites, but also effectively buffers the volume variation during repeated charge–discharge processes, which is highly favorable for enhancing the reaction kinetics and cycling stability of the electrode.

**Fig. 4:**
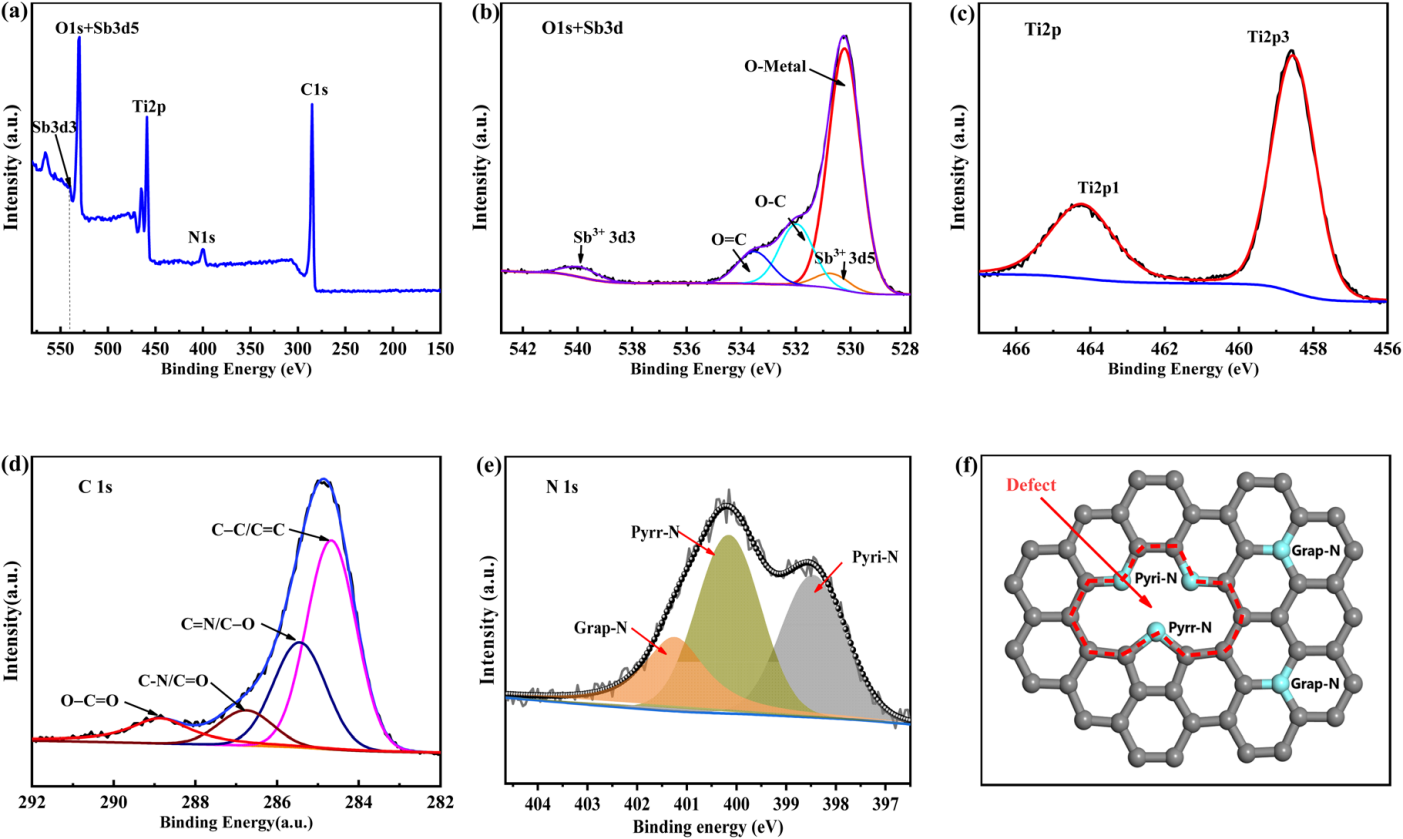
(a) XPS full spectrum of the Sb–TiO_2_/C NFs. (b) XPS high-resolution spectrum of Sb 2p, (c) Ti 2p, (d) C 1s and (e) N 1s for the Sb–TiO_2_/C NFs. (f) Schematic of the graphitic N, pyrrolic N, and pyridinic N in Sb–TiO_2_/C NFs.

Aiming at a comprehensive understanding of the surface chemistry of as-electrospun samples, X-ray photoelectron spectra are collected and shown in Fig. 4a. Ti, O, Sb, C, and N elements that were identified by the XPS full spectrum of Sb–TiO_2_/C NF. These elements are probably derived from TPIP, SbCl_3,_ and PVP, respectively. In the high-resolution O 1s and Sb 3d spectrum of Sb–TiO_2_/C NFs (Fig. 4b), due to the direct overlap between O 1s and Sb 3d_5/2_, the peak of Sb3d3/2 (situated at 539.9 eV) was utilized as a guide for peak fitting. Among the fitting peaks, any peak could not be appointed to antimony metal (Sb^0^) that is identified by its XRD patterns. Nevertheless, Sb^3+^ species could be found (539.9 and 530.6 eV). This absence of Sb^0^ may be attributed to surface oxidation of Sb metal. Generally, Sb metal is oxidized in the air naturally and coated with a thin layer of antimony oxide_S_, which is proofed by the XPS spectrum of antimony metal in the previous literature.^36^ In the fact that there is very few Sb in the matrix, merely the superficial Sb^3+^ could be found in the XPS spectrum. There existed three types of O species, situated at 533.4 eV, 531.9 eV, 530.2 eV, which could be assigned to O=C, O–C, and O–metal, respectively. The two peaks at 545.8 eV (Ti 2p_3/2_), and 464.2 eV (Ti 2p_1/2_) in Fig. 4C demonstrated the valence state of Ti element is Ti^2+^. As shown in Fig. 4d, the high-resolution spectrum of C1s can be deconvolved into four species, that is, the peak located at 284.8 eV for the C–C/C=C species, 285.4 eV for C=N/C–O, 286.8 eV for C–N/C=O, and 288.8 eV for O–C=O (carboxylates). Based on the presence of C–O species and C–N species in the sample, N atoms were probably doped in the carbon matrix. N 1s high-resolution spectrum (Fig. 4f) additionally demonstrated that N atoms had been doped in the carbon matrix, resulting in three nitrogen species that are graphene-N, pyrrole-N, and pyridine-N situated at 401.3 eV, 400.1 eV, and 398.4 eV respectively.^37,38^ These self-doped Nitrogen atoms could not only provide plenty of electrons to conjugate with the delocalized π system of carbon matrix but also render numerous defects for the carbon matrix, as is illustrated with a dotted circle in Fig. 4f. These defects could impart substantial active sites for Li^+^/Na^+^ storage.

**Fig. 5:**
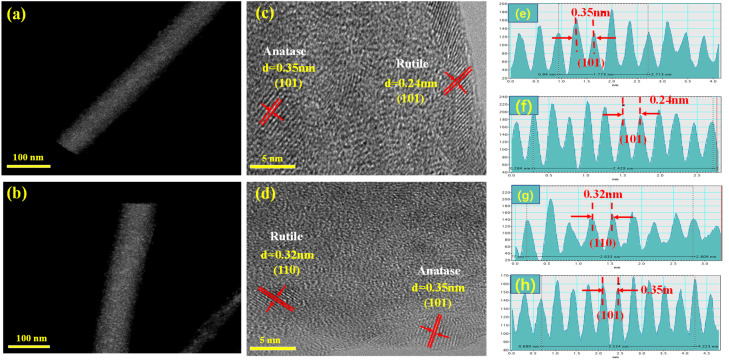
High-angle annular dark field (HAADF) images of TiO_2_/C NFs (a) and Sb–TiO_2_/C NFs (b); HR-TEM images of TiO_2_/C NFs (c) and Sb–TiO_2_/C NFs (d); interplanar crystal spacing statistical tables of TiO_2_/C NFs (e and f) and Sb–TiO_2_/C NFs (g and h).

High-Angle Annular Dark Field (HAADF) microscopy and High-Resolution Transmission Electronic Microscope (HR-TEM) were employed to estimate the micromorphology of the as-electrospun samples. The HAADF images (Fig. 5a and b) proved that TiO_2_/C and Sb–TiO_2_/C samples have a nanofibers microstructure with a diameter of approximately 140 nm and 160 nm; Fig. 5c and d showed HR-TEM images of samples,^3–5,39^ in which clear and palpable lattice fringes were marked with short red lines. In the HR-TEM image of TiO_2_/C NFs, the distinct lattice fringes with a *d*-spacing of 0.35 and 0.24 nm could be ascribed to (101) face of anatase and (101) face of rutile, respectively. The corresponding interplanar crystal spacing statistical tables are shown in Fig. 5e and f. This result complied well with the XRD patterns (Fig. 3a). Despite its poor crystallinity (see XRD patterns in Fig. 3b), the lattice fringes of the anatase phase with a *d*-spacing of 0.35 nm (101) were also found in Sb–TiO_2_/C NFs, as shown in Fig. 5d and h. However, the distinct lattice fringes with a *d*-spacing of 0.32 nm should be ascribed to (110) face of the rutile phase (Fig. 5g), which is slightly different from the (101) phase of TiO_2_/C NFs. It is likely due to the introduction of Sb atoms, which led to the partial phase transformation from anatase to rutile and promoted the preferential growth of the rutile (110) plane. Moreover, the lattice fringes of Sb metal could not be found at all, which might be attributed to its limited contents and small size of Sb nanoparticles or being covered with the lattice fringes of titanium.

**Fig. 6:**
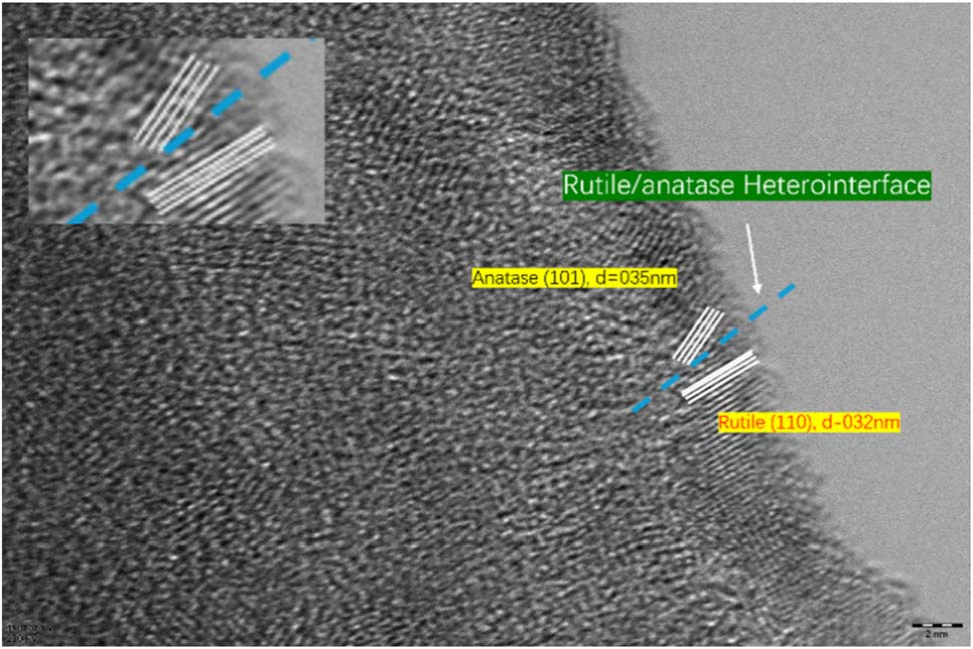
High-resolution TEM image of the Sb–TiO_2_/C nanofibers, showing the clear rutile/anatase heterointerface. The inset shows the magnified view of the interface region, with lattice fringes corresponding to anatase (101, *d* = 0.35 nm) and rutile (110, *d* = 0.32 nm) phases.

To further directly visualize the rutile/anatase heterointerface, we conducted supplementary high-resolution TEM characterization on the Sb–TiO_2_/C nanofibers, with the results shown in Fig. 6. The image clearly captures two sets of distinct, indexable and tightly adjacent lattice fringes in the same nanoscale field of view: the lattice spacing of 0.35 nm corresponds to the (101) crystal plane of anatase TiO_2_, and the lattice spacing of 0.32 nm corresponds to the (110) crystal plane of rutile TiO_2_. A continuous heterointerface between the two phases is clearly observed without obvious gaps or amorphous transition regions, which provides direct visual evidence for the successful construction of the rutile/anatase heterojunction.

The uniform distribution of Ti, O and C elements throughout the continuous nanofiber is further verified by HAADF-STEM elemental mapping (Fig. S7, SI), which confirms the homogeneous coupling of anatase and rutile phases at the nanoscale, ruling out the possibility of simple physical mixing of two separate phases. Moreover, the uniform distribution of the weak but detectable Sb signal across the TiO_2_ and carbon matrix confirms the homogeneous incorporation of Sb dopants at the micro-scale.

### 3.3 Electrochemical performance and reaction mechanism

**Fig. 7:**
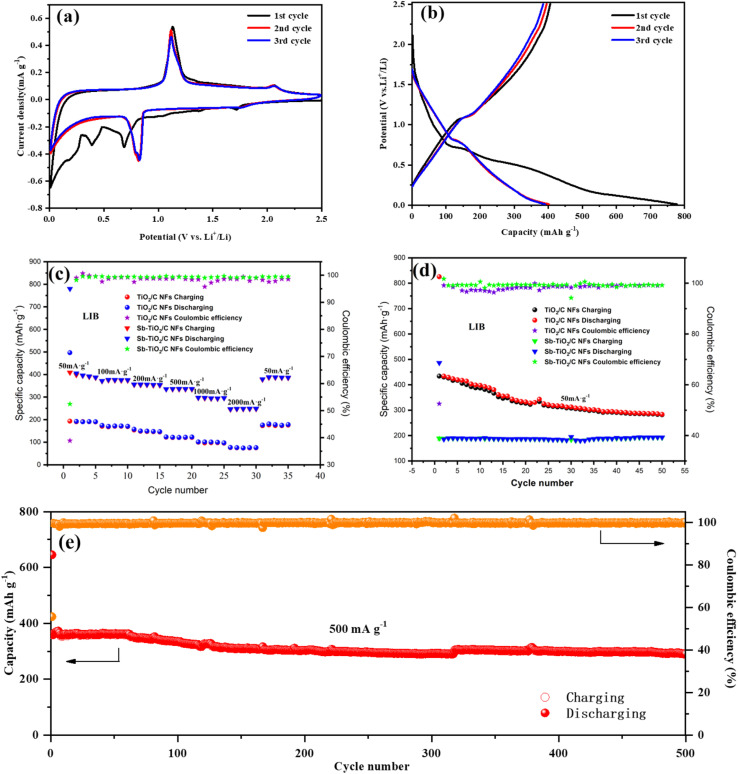
Electrochemistry performance of the LIBs using Sb–TiO_2_/C nanofibers paper as working electrode and Li metal as the counter/reference electrode. (a) Cyclic voltammetry curve for the first three cycles between 0.01 and 2.5 V with a scan rate of 0.1 mV s^−1^ (b) discharge–charge voltage profiles at a rate of 50 mA g^−1^ (c) rate performance at various current densities (50–2000 mA g^−1^). (d) Cycling performance at a current density of 50 mA g^−1^. (e) Long-term cycling performance (500 cycles) for the Sb–TiO_2_/C NFs paper anode at the potential window of 0.01 to 2.5 V (*vs.* Na^+^/Na) with the scan rate of 0.1 mV s^−1^, using Sb–TiO_2_/C nanofiber paper as the working electrode.

Lithium storage performance was tested in coin-type half cells, directly using the as-electrospun nanofibers paper as freestanding working anodes without any binder, conductive additives, current collector, and slurry-casting process, which could reduce the electrode weight and thus enhance the energy/power densities of full cells. Cyclic voltammetry (CV) was performed in the first three cycles at the potential window of 0.01 to 2.5 V (*vs.* Li^+^/Li) with a scan rate of 0.1 mV s^−1^, of which curves showed in Fig. 7a. The reduction peak at 1.72 and 0.83 V could be assigned to the lithiation reaction from Sb to Li_3_Sb and that from TiO_2_ to Li_*x*_TiO_2_.^40,41^ The anodic peaks centered at 1.13 and 2.06 V probably originate from the delithiation reactions of Li_3_Sb and Li_*x*_TiO_2_.^42^ In the initial scan, reductive peaks at 0.68, 0.39 V, and below can be clearly found and thoroughly disappear in the next two cycles. This is easily understood and attributed to the irreversible formation of solid electrolyte interface (SEI), which is extremely common and inevitable in LIBs or SIBs and would induce irreversible capacity loss. The cyclic voltammograms of the third cycle overlap well with the second one, indicating excellent stability of the half coin cell. Quantitatively, the potential difference (Δ*E*_p_) between the lithiation peak of TiO_2_ (0.83 V) and the corresponding delithiation peak (2.06 V) of the Sb–TiO_2_/C electrode is 1.23 V, which is 138 mV lower than that of the pristine TiO_2_/C electrode (1.368 V). This significant reduction of Δ*E*_p_ directly proves that the Sb doping and rutile/anatase heterojunction construction effectively reduce the electrochemical reaction polarization and charge transfer impedance of the electrode, confirming the accelerated electrochemical reaction kinetics. To further quantitatively investigate the charge transfer kinetics of the electrode, electrochemical impedance spectroscopy (EIS) was performed after the initial cycle, and the data was fitted using an equivalent circuit (Fig. S8). The fitted curve shows excellent agreement with the experimental data (*χ*^2^ = 0.0029), indicating the suitability of the model. The key fitted parameters reveal a low solution resistance (*R*_s_ = 3.49 Ω) and a notably low charge-transfer resistance (*R*_ct_ = 70.58 Ω). This *R*_ct_ value is significantly lower than that reported recently for electrospun TiO_2_/C nanofiber electrodes (*R*_ct_ = 155 Ω).^43^ This quantitative comparison demonstrates that the optimized 1D carbon network constructed *via* our preparation process greatly reduces the interface charge transfer barrier, thereby endowing the electrode with superior intrinsic reaction kinetics. The first three discharge–charge curves at a rate of 50 mA g^−1^ are shown in Fig. 7b. Based on the full mass of Sb–TiO_2_/C nanofibers, the first discharge and charge capacities were calculated as 782 mA h g^−1^ and 408 mA h g^−1^, respectively. The large initial capacity loss (47.8%) is primarily ascribed to the formation of SEI films and partially irreversible intercalation of Li^+^ into the carbon nanofibers. During the subsequent discharge–charge cycles, the curves are almost overlapped implying good cyclability and the minimized polarization for the electrode. Meanwhile, the charge–discharge voltage platform hysteresis of the Sb–TiO_2_/C electrode is only 96 mV at 50 mA g^−1^, which is far smaller than that of the pristine TiO_2_/C electrode (214 mV). The smaller voltage hysteresis further verifies the faster charge transfer kinetics and higher electrochemical reversibility of the Sb–TiO_2_/C electrode, which is consistent with the Δ*E*_p_ analysis results of CV curves. Fig. 7c presented the galvanostatic rate capability of the as-synthesized nanofibers in LIBs. Sb-doped and undoped TiO_2_/C nanofibers are used as electrodes to compare their rate performance at various current densities. As seen from Fig. 7c, Sb-doped nanofibers show much better rate performance compared with the undoped. The specific capacity slowly decreases when current density increases from 50 to 2000 mA g^−1^, while when the current density is set back to 50 mA g^−1^, the specific capacity restores almost identical to that of the second cycle. Sb-doped TiO_2_/C nanofibers show excellent rate performance. The discharge specific capacity keeps 430 mA g^−1^ at the current density of 50 mA g^−1^, even at the high current density of 2000 mA g^−1^, the discharge specific capacity remains 228 mA g^−1^ (77 mA g^−1^for undoped TiO_2_/C NFs). The rate performance of different addition amounts of Sb is showed in Fig. S9a, demonstrating that the addition of Sb could extensively improve the rate performance, which was also asserted in the recent similar literature.^15^Fig. 7d showed the cycling performance of the half coin cells at a low current density of 50 mA g^−1^ for 50 cycles. Sb–TiO_2_/C nanofibers also deliver higher specific capacity than undoped TiO_2_/C nanofibers (280 mA g^−1^ and 192 mA g^−1^ after 50 cycles, respectively). The Sb–TiO_2_/C NFs paper electrode declined slowly in specific capacity with the increasing of the cycle number and kept almost constant after the 40th cycle, demonstrating its good cycle stability. The corresponding electrochemistry performance with different amounts of Sb was illustrated in Fig. S9b. Moreover, long-term cycling performance was investigated at 500 mA g^−1^ for 500 contiguous cycles (see Fig. 7e). It is noted that the capacity in the initial 10 cycles is close to that obtained at 50 mA g^−1^, which can be ascribed to the sufficient electrochemical activation and stable SEI formation of the self-standing electrode in the early cycles. The Sb–TiO_2_/C electrode exhibits extremely high cycling stability, even keep a discharge capacity of 291.2 mA h g^−1^ after 500 cycles. The retention rate is 80.8% based on the second cycle. It should be noted that the average decay rate is ultra-low, about 0.038% per cycle during the 500 cycles. Apart from the first cycle, the coulombic efficiency keeps above 99%, indicating its excellent reversibility in the delithiation/lithiation process.
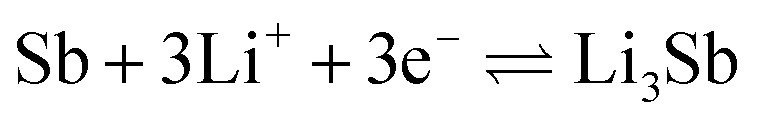

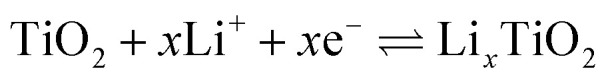


The excellent rate performance and long-cycle stability are direct manifestations of the accelerated ion/electron transfer kinetics of the Sb–TiO_2_/C electrode. Even at an ultra-high current density of 2000 mA g^−1^, the electrode still maintains a high reversible specific capacity of 228 mA h g^−1^ for LIBs, which is 3 times that of the pristine TiO_2_/C electrode (77 mA h g^−1^). The same kinetic enhancement effect is also reflected in the sodium storage performance: the electrode still delivers a reversible capacity of 162 mA h g^−1^ at 2000 mA g^−1^ for SIBs, further confirming the universality of the kinetic optimization strategy in this work. The significant improvement of reaction kinetics is attributed to the synergistic effect of three structural design strategies: (1) the rutile/anatase heterojunction constructs a built-in electric field at the phase interface, which reduces the diffusion energy barrier of Li^+^/Na^+^ and accelerates the interfacial charge transfer, which is consistent with the kinetic enhancement mechanism of anatase/rutile TiO_2_ homojunction verified in previous reports; (2) the 1D porous nanofiber structure with intercrossed network shortens the ion/electron transport path, provides sufficient contact area between the electrode and electrolyte, and promotes rapid ion diffusion; (3) the N self-doping in the carbon matrix introduces abundant defect sites, which not only provides additional active sites for ion storage, but also improves the intrinsic conductivity of the carbon matrix, further accelerating electron transport. The initial coulombic efficiency (ICE) of the Sb–TiO_2_/C electrode is ∼38.9% for LIBs. The relatively low ICE is mainly attributed to three factors: (1) the irreversible formation of SEI film on the large specific surface area (153.8 m^2^ g^−1^) of the nanofibers; (2) the irreversible Li^+^ adsorption at the defect sites of the N-doped carbon matrix; (3) the partial irreversible alloying reaction of Sb during the first discharge process. Notably, the coulombic efficiency rapidly rises to over 99% after the first 5 cycles and remains stable during the subsequent 500 cycles, confirming the excellent stability of the formed SEI film. Feasible strategies to optimize the ICE include pre-lithiation of the freestanding electrode, electrolyte formula optimization, and surface coating modification of the nanofibers.

**Fig. 8:**
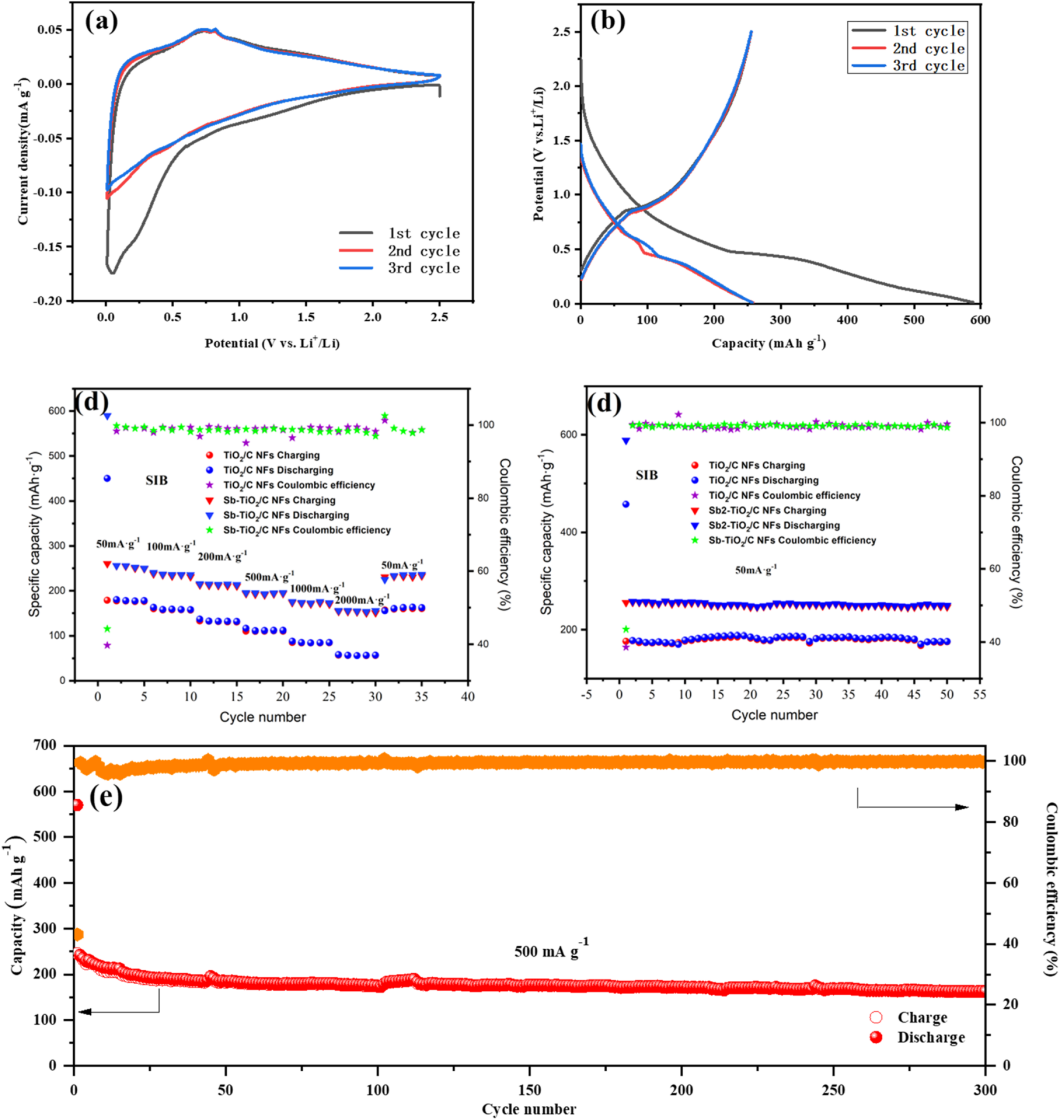
Electrochemistry performance of the SIBs using Sb–TiO_2_/C nanofibers paper as working electrode and Na metal as the counter/reference electrode respectively (a) Cyclic voltammetry curves between 0.01 and 2.5 V with a scan rate of 0.1 mV s^−1^. (b) Discharge–charge voltage profiles at a rate of 50 mA g^−1^. (c) Rate performance at various current densities (50–2000 mA g^−1^). (d) Cycling performance at a current density of 50 mA g^−1^. (e) Long-term cycling performance (300 cycles), using Sb–TiO_2_/C nanofiber paper as the working electrode.

Because of the outstanding performance in LIBs, the electrochemistry performance of the as-electrospun NFs used as anode in SIBs was also investigated in coin-type half cells without any slurry-casting process, as shown in Fig. 8. As is seen in Fig. 8a (the first cathodic scan), an irreversible peak appeared over a wide potential range from 0.1 to 0.7 V *versus* Na^+^/Na, which corresponded to the formation of SEI film and Na_*x*_Sb. This peak disappeared during the subsequent two cycles. The anodic peaks around 0.70 V and 0.82 V could be assigned to the delithiation reactions of Na_3_Sb and Na_*x*_TiO_2_.^44,45^ The third CV curve overlapped well with the second one, which demonstrated its good stability. Fig. 8b shows the galvanostatic discharge–charge curves of the Sb–TiO_2_/C NFs anode at a rate of 50 mA g^−1^ for the first three cycles. Like LIBs, the capacity loss of the first discharge in SIBs might originate from the irreversible SEI films in the first discharge–charge cycle. Almost overlapped discharge–charge curves are observed, indicating that outstanding cycling stability and minimal polarization for Sb–TiO_2_/C NFs anode. Fig. 8c shows the galvanostatic rate capability of the as-synthesized nanofibers in SIBs. When current density increases from 50 to 2000 mA g^−1^, the specific capacity slowly decreases. Nevertheless, when the current density is set back to 50 mA g^−1^ the specific capacity restores nearly identical to that of the second cycle, which is crucial for the applications of high-power SIBs. When Sb-doped nanofibers and undoped nanofibers are concerned, the former shows much better rate performance compared with the latter. The discharge specific capacity of SIBs with Sb-doped nanofibers shows about 230 mA g^−1^ at the current density of 50 mA g^−1^, while it remains 162 mA g^−1^. The addition of Sb could better the performance rate of SIBs, which is demonstrated with the rate performance experiments under different addition amounts of Sb (Fig. S9c and d). Fig. 8d demonstrates the cycling performance of SIBs at a low current density of 50 mA g^−1^ for 50 cycles. Sb–TiO_2_/C nanofibers also deliver higher specific capacity than undoped TiO_2_/C nanofibers (about 250 mA g^−1^ and 180 m mA g^−1^ after 50 cycles, respectively). Unlike in the case of LIBs, both the Sb–TiO_2_/C NFs and undoped TiO_2_/C NFs electrode almost kept constant in specific capacity with the increasing of the cycle number, demonstrating its excellent cycle stability for SIBs.

The above excellent half-cell electrochemical performance, including high reversible specific capacity, ultra-long cycling stability and outstanding rate capability, lays a solid foundation for the application of this flexible self-standing anode in full-cell systems. Notably, the as-prepared Sb–TiO_2_/C nanofiber paper exhibits a stable working voltage window of 0.01–2.5 V (*vs.* Li^+^/Li and Na^+^/Na), which is highly compatible with the mainstream commercial cathodes for LIBs (LiFePO_4_, LFP) and SIBs (Na_3_V_2_(PO_4_)_3_, NVP). Meanwhile, the binder-free and current collector-free integrated structure can effectively simplify the assembly process of flexible full-cells and avoid the negative impact of inactive components on the overall energy density of the device. The relevant research on the matching of this anode with LFP/NVP cathodes and the electrochemical performance optimization of flexible full-cells will be systematically carried out in our follow-up work. Long-term cycle stability of the Sb–TiO_2_/C NFs electrode for SIBs was evaluated through the galvanostatic discharge–charge experiments executed at a higher current density of 500 mA g^−1^. The result is shown in Fig. 8e. After 300 cycles, the Sb–TiO_2_/C NFs electrode remains a capacity of 162 mA g^−1^. Unexpectedly, the Coulomb efficiency of the first discharge is only 43%, which is probably attributed to the formation of the irreversible SEI membrane. However, the Coulomb efficiency rises to nearly 99% after the first several cycles. The electrochemical performance in SIBs, while still good, is lower than in LIBs, which is probably attributed to the larger radius of Na ions, the slower transport efficiency of Na ions, and the larger volume change during charge and discharge.^46–48^

For SIBs, the ICE of the electrode is %, which is lower than that of LIBs. This is mainly due to the larger ionic radius of Na^+^, which leads to more severe irreversible side reactions and a more unstable SEI film during the first cycle, which is a common phenomenon for most anode materials for SIBs. The coulombic efficiency also rises to nearly 99% after the first several cycles and remains stable during the subsequent 300 cycles, verifying the good structural stability of the electrode. Feasible strategies to further optimize the initial coulombic efficiency include pre-lithiation/pre-sodiation treatment of the free-standing electrode, electrolyte formula optimization with functional film-forming additives, and surface coating modification of the nanofibers to reduce irreversible side reactions on the material surface.

**Fig. 9:**
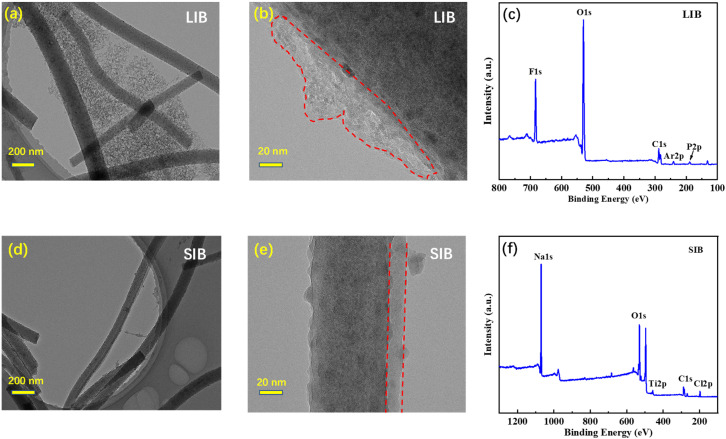
TEM images and full XPS spectra of the cycled electrodes in LIBs and SIBs. (a) TEM image of the LIBs electrode after 500 cycles with a 200 nm scale bar. (b) TEM image of the LIBs electrode after 500 cycles with a 20 nm scale bar. (c) Full XPS spectrum of the LIBs electrode after 500 cycles. (d) TEM image of the SIBs electrode after 300 cycles with a 20 nm scale bar. (e) TEM image of the SIBs electrode after 300 cycles with a 20 nm scale bar. (f) Full XPS spectrum of the SIBs electrode after 300 cycles.

To gain insight into the mechanism for the excellent cycling stability of the Sb–TiO_2_/C NFs electrode, the electrodes were tear down from the cycled LIBs and SIBs and rinsed with dimethyl carbonate (DMC) to remove the residues on it. Fig. 9a, c, and 9b and d show the TEM images of the after-cycled electrode in LIBs and SIBs, respectively. The after-cycled Sb–TiO_2_/C NFs electrodes still hold their original structure both in LIBs and SIBs (Fig. S10 and S11), suggesting that the electrode structure can effectively accommodate the volume change during cycling. The TEM images in Fig. 9b and e elucidate that the Sb–TiO_2_/C NFs were covered by an SEI film (circled with red dotted lines) on their surface. The SEI film is mainly formed in the first cycle, which might be the reason accounting for the low initial coulombic efficiency. Interestingly, the electrode surface of SIBs was somewhat smoother than that of LIBs, which is probably due to the addition of FEC when fabricating the sodium half-cell. Besides, Fig. 9c and f are XPS full spectra of the after-cycled electrode in LIBs and SIBs, respectively. Fluorine, chlorine, phosphorus, sodium, *etc.* are made up of the SEI membrane, which is in line with previous studies.^49,50^ Notably, the complete nanofiber structure maintained after long-term cycles directly confirms that the 3D intercrossed porous carbon matrix can effectively alleviate the volume expansion of Sb during the alloying/dealloying process, avoiding the pulverization of active materials, which is consistent with the volume expansion mitigation mechanism of alloy-type anodes revealed in previous reports.^26^ Meanwhile, the stable SEI film formed on the nanofiber surface after the initial cycle can effectively avoid the continuous consumption of electrolyte, which is the key to the excellent long-cycle stability of the electrode.^27^ The above synergistic effect also solves the common problem of poor structural stability of flexible alloy-based anodes during repeated bending and cycling, further verifying the application potential of this material in flexible energy storage devices.^28^

## 4. Conclusions

In summary, flexible self-standing Sb–TiO_2_/C nanofiber papers were successfully fabricated *via* electrospinning and carbonization. Moderate Sb doping optimizes the porous structure and induces the construction of a rutile/anatase heterojunction, which significantly enhances charge transfer kinetics and provides abundant active sites for Li^+^/Na^+^ storage. Benefiting from the carbon confinement effect and robust nanofiber framework, the binder-free electrode exhibits excellent mechanical flexibility and outstanding structural stability upon long-term cycling. Consequently, the electrode delivers a stable capacity of 291.2 mA h g^−1^ after 500 cycles at 500 mA g^−1^ for LIBs and 162 mA h g^−1^ after 300 cycles for SIBs. This work confirms that Sb-modified TiO_2_/C nanofibers are competitive anode candidates for flexible lithium- and sodium-ion batteries. Further investigations on full-cell matching and in-depth kinetic characterization will be conducted to advance the practical application of this material.

## Author contributions

Kun Su: conceptualization, methodology, formal analysis, writing – original draft. Jinhua Dai: funding acquisition, writing – review & editing. All authors have read and agreed to the published version of the manuscript.

## Conflicts of interest

The authors declare that they have no known competing financial interests or personal relationships that could have appeared to influence the work reported in this paper.

## Supplementary Material

RA-016-D6RA02199A-s001

## Data Availability

All relevant data supporting the findings of this study are openly available and can be accessed from the corresponding author upon reasonable request. The data include the original experimental data of Sb–TiO_2_/C nanofiber paper synthesis, characterization data (*e.g.*, morphology, crystal structure), and electrochemical test data (*e.g.*, charge–discharge curves, cycling stability, rate performance) that are essential for reproducing the results reported in the manuscript. Supplementary information (SI) is available. See DOI: https://doi.org/10.1039/d6ra02199a.
