# Supplementary material for: Sb–TiO2/C nanofiber paper as a flexible high-performance anode for lithium-ion and sodium-ion batteries
Source: RSC Adv. 2026 May 5;16(25):23058–70. doi: 10.1039/d6ra02199a (PMC13142657; doi:10.1039/d6ra02199a)
Supplement: RA-016-D6RA02199A-s001 [file RA-016-D6RA02199A-s001.pdf]

Supplementary Information (SI) for RSC Advances.
This journal is © The Royal Society of Chemistry 2026

Supporting Information

Sb-TiO_2/C composite nanofibers paper as high-performance anode materials for Lithium and Sodium Ion Batteries

Table of contents

Fig.S1. Optical photographs of (a) precursor (b) stabilized and (c) carbonized nanofiber membrane (1ml TTIP and 0.2 g $SbCl_3$..2

Fig. S2. The SEM image of Sb-TiO_2/C (0.4 g $SbCl_3$) nanofibers, showing plenty of aggregative plots..2

Fig. S3 Digital photographs of the as-prepared folded and unfolded Sb-TiO_2/C nanofiber paper: (a) the paper after multiple folding; (b) the paper after unfolding, showing its intact structure. 3

Fig. S4. Raman Fitting curves of TiO_2/C NFs (a)and TiO_2-Sb/C NFs(b) 3

Fig. S5 Raman spectra (0–2000 cm^{-1}) of TiO_2/C and Sb-TiO_2/C NFs including the fingerprint region below 1000 cm^{-1}. 4

Fig. S6. N_2 adsorption-desorption isotherm (insets) and BJH Pore size distribution curve of (a) TiO_2/C NFs, (b) Sb(0.2)-TiO_2/C NFs and Sb(0.4)-TiO_2/C NFs 4

Fig. S7.Electrochemical impedance spectroscopy (EIS) Nyquist plot of the TiO_2/C nanofiber NFs electrode at 50 $mA \cdot g^{-1}$ after the first cycle in LIBs (the inset part is the equivalent circuit model). 5

Fig. S8. TEM images of TiO_2/C NFs and Sb-TiO_2 /C NFs 6

Fig. S9. SEM images After long-term cycling 6

Fig. S10. Rate performance and cycling performance of TiO_2/C with different addition amounts of Sb 7

Table S1. Analysis of the content of Sb and Ti in Sb-TiO_2/C NFs by ICP-OES 8

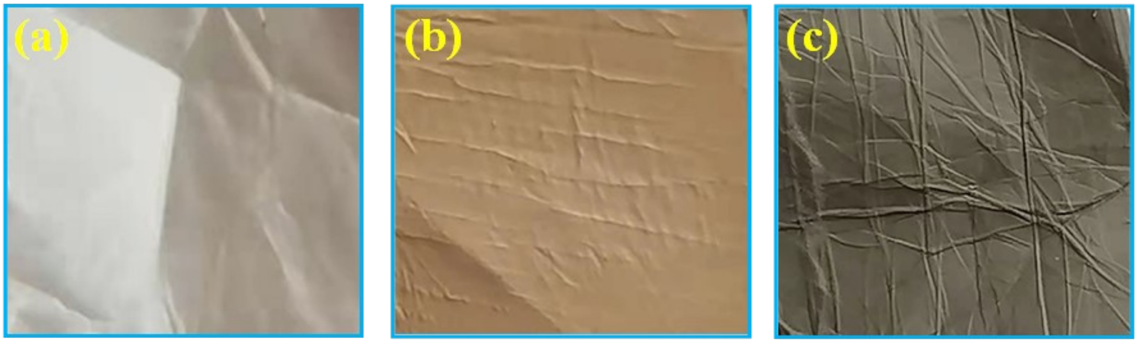

Fig.S1. Optical photographs of (a) precursor (b) stabilized and (c) carbonized nanofiber membrane (1ml TTIP and 0.2 g $SbCl_3$

Fig. S2. The SEM image of Sb-TiO_2/C (0.4 g $SbCl_3$) nanofibers, showing plenty of aggregative plots

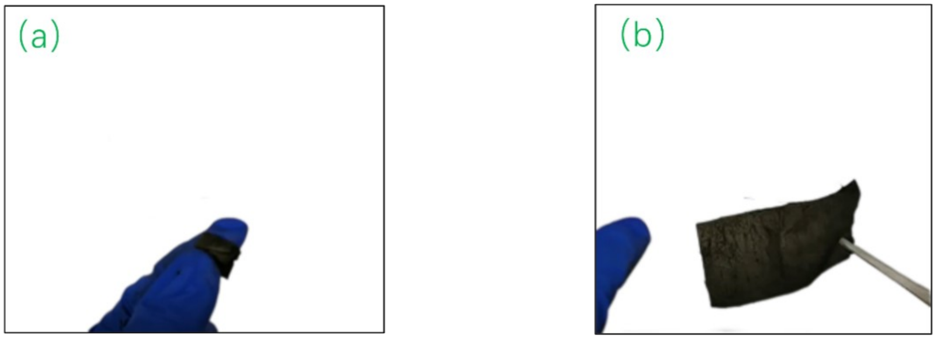

Fig. S3 Digital photographs of the as-prepared folded and unfolded Sb-TiO_2/C nanofiber paper: (a) the paper after multiple folding; (b) the paper after unfolding, showing its intact structure.

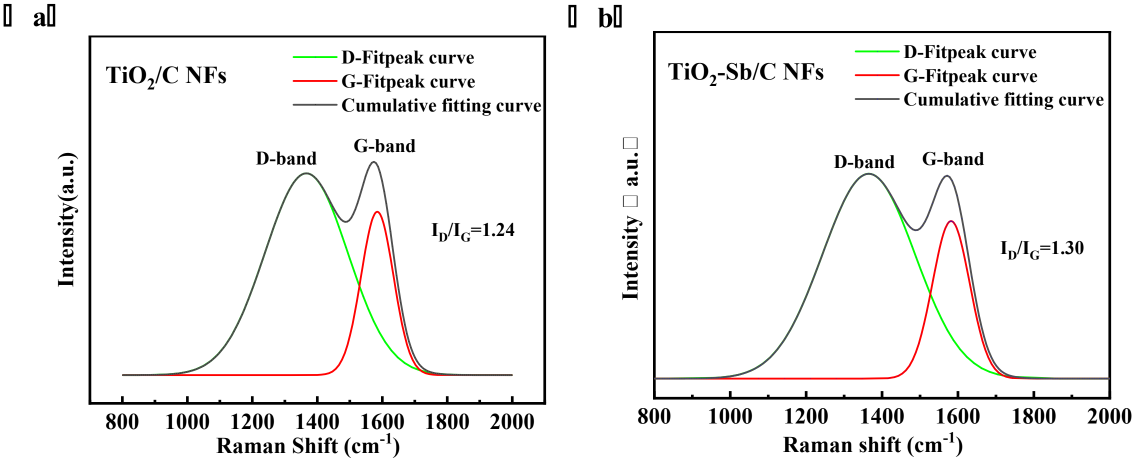

Fig. S4. Raman Fitting curves of TiO_2/C NFs (a)and TiO_2-Sb/C NFs(b)

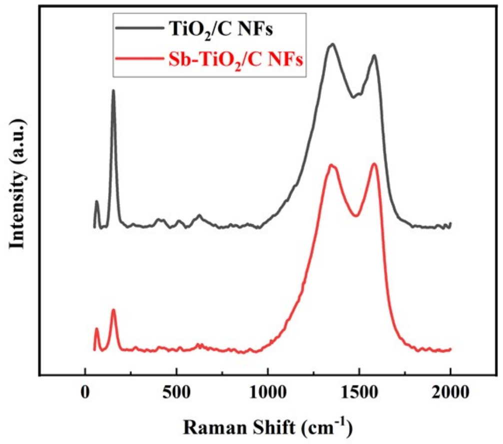

Fig. S5 Raman spectra (0–2000 cm^{-1}) of TiO_2/C and Sb-TiO_2/C NFs including the fingerprint region below 1000 cm^{-1}.

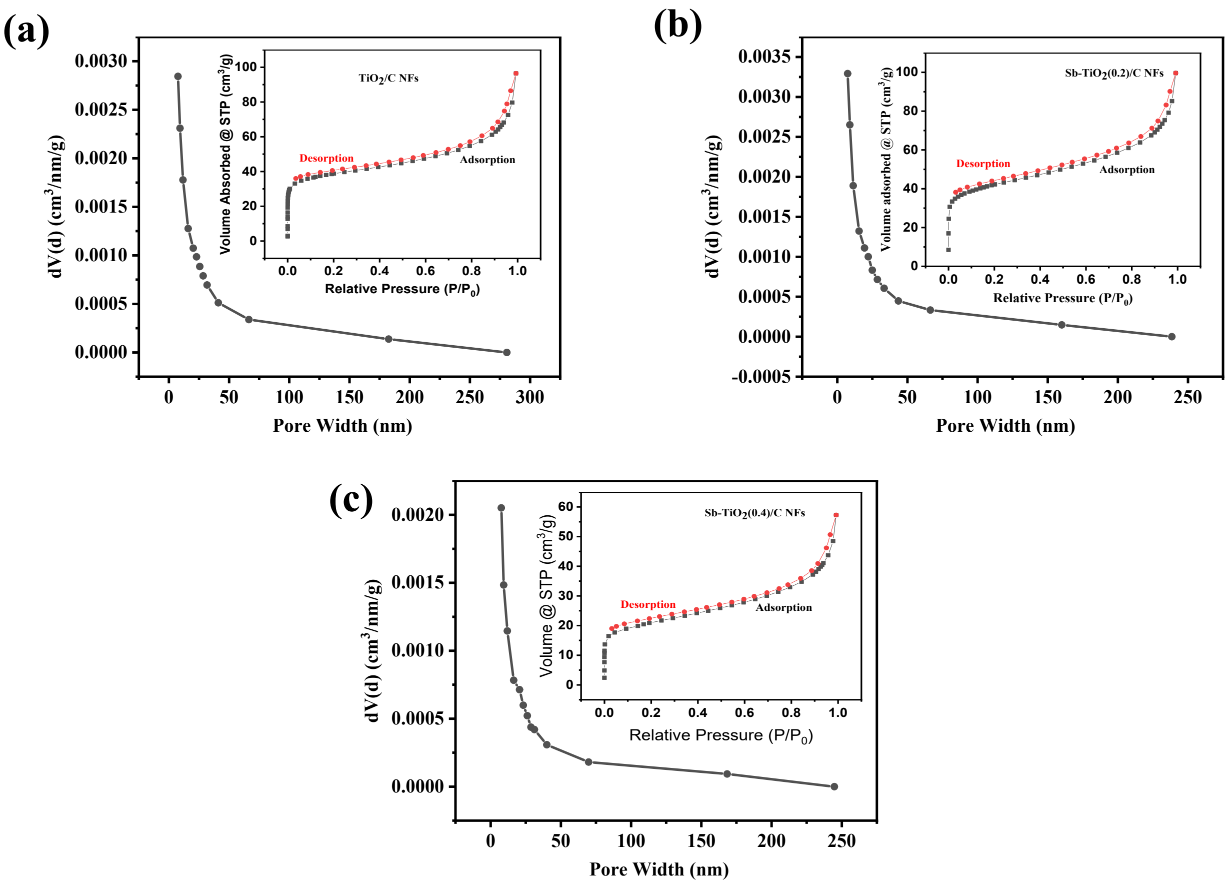

Fig. S6. N_2 adsorption-desorption isotherm (insets) and BJH Pore size distribution curve of (a) TiO_2/C NFs, (b) Sb(0.2)-TiO_2/C NFs and Sb(0.4)-TiO_2/C NFs

Fig. S7. EDS elemental mapping of the Sb-TiO_2/C nanofibers. (a) SEM image (electron image) of a representative nanofiber junction. Corresponding elemental distribution maps for (b) C k α 1, (c) Ti K α 1, and (d) O K α 1. The uniform distribution of the weak but detectable Sb signal (highlighted in orange) across the TiO_2 and carbon matrix confirms the homogeneous incorporation of Sb dopants at the micro-scale. The low intensity of the Sb signal is consistent with its designed low doping concentration, as further definitively confirmed by the surface-sensitive XPS analysis in Fig. 4b of the main text.

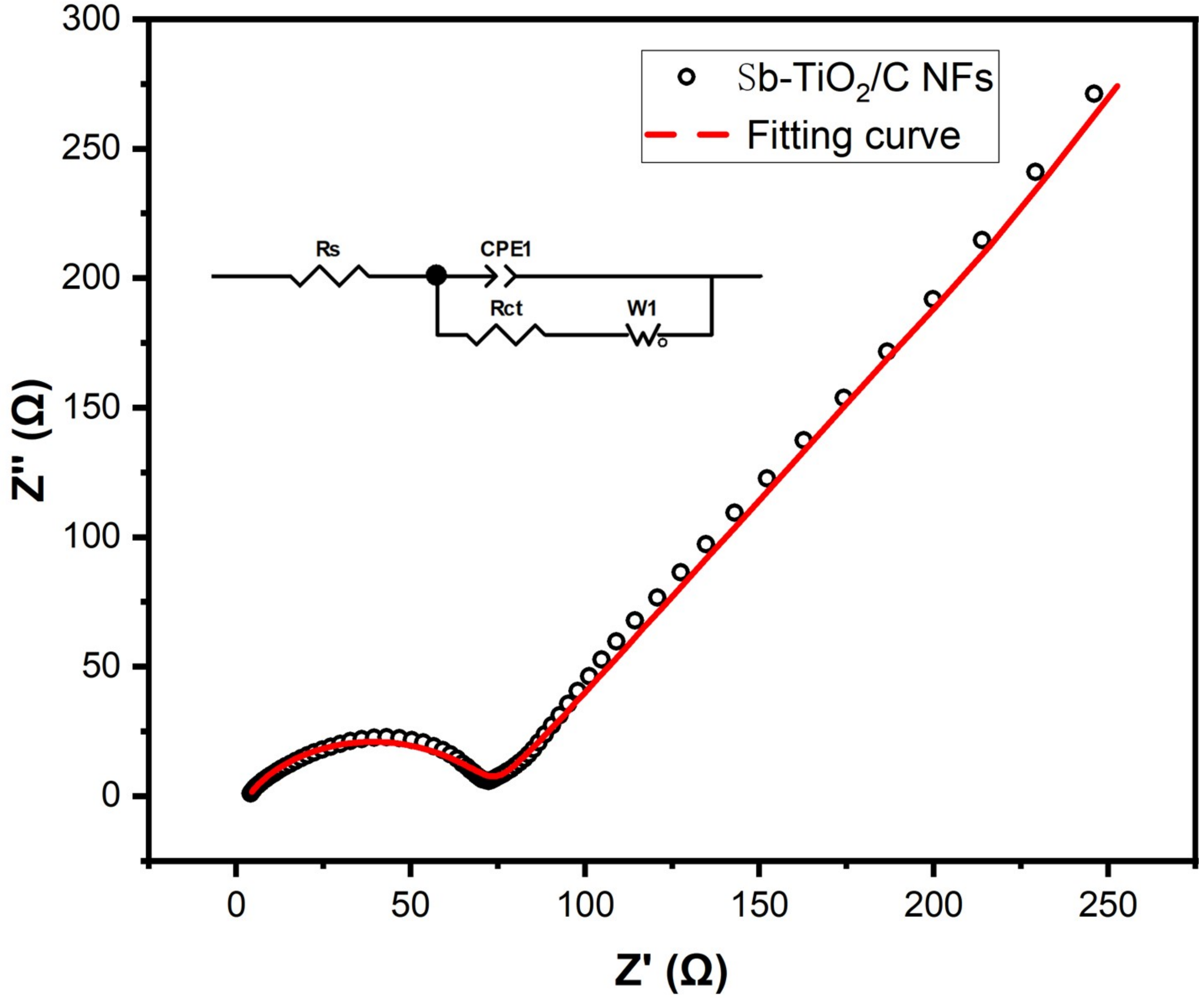

Fig. S8. Electrochemical impedance spectroscopy (EIS) Nyquist plot of the TiO_2/C nanofiber NFs electrode at 50 $mA \cdot g^{-1}$ after the first cycle in LIBs (the inset part is the equivalent circuit model).

Fig. S9. Rate performance and cycling performance of TiO_2/C with different addition amounts of Sb

(a) Rate performance at various current densities from 50mA·g^{-1} to 2000 mA·g^{-1} in LIBs. (b) Cycling performance at a current density of 50mA g^{-1} in LIBs. (c) Rate performance at various current densities from 50mA·g^{-1} to 2000 mA·g^{-1} in SIBs. (d) Cycling performance at a current density of 50mA g^{-1} in SIBs.

Note: The addition amount of Sb is 0g, 0.2g, 0.4g for TiO_2/C NFs, Sb2-TiO_2/C NFs, Sb4-TiO_2/C NFs, respectively.

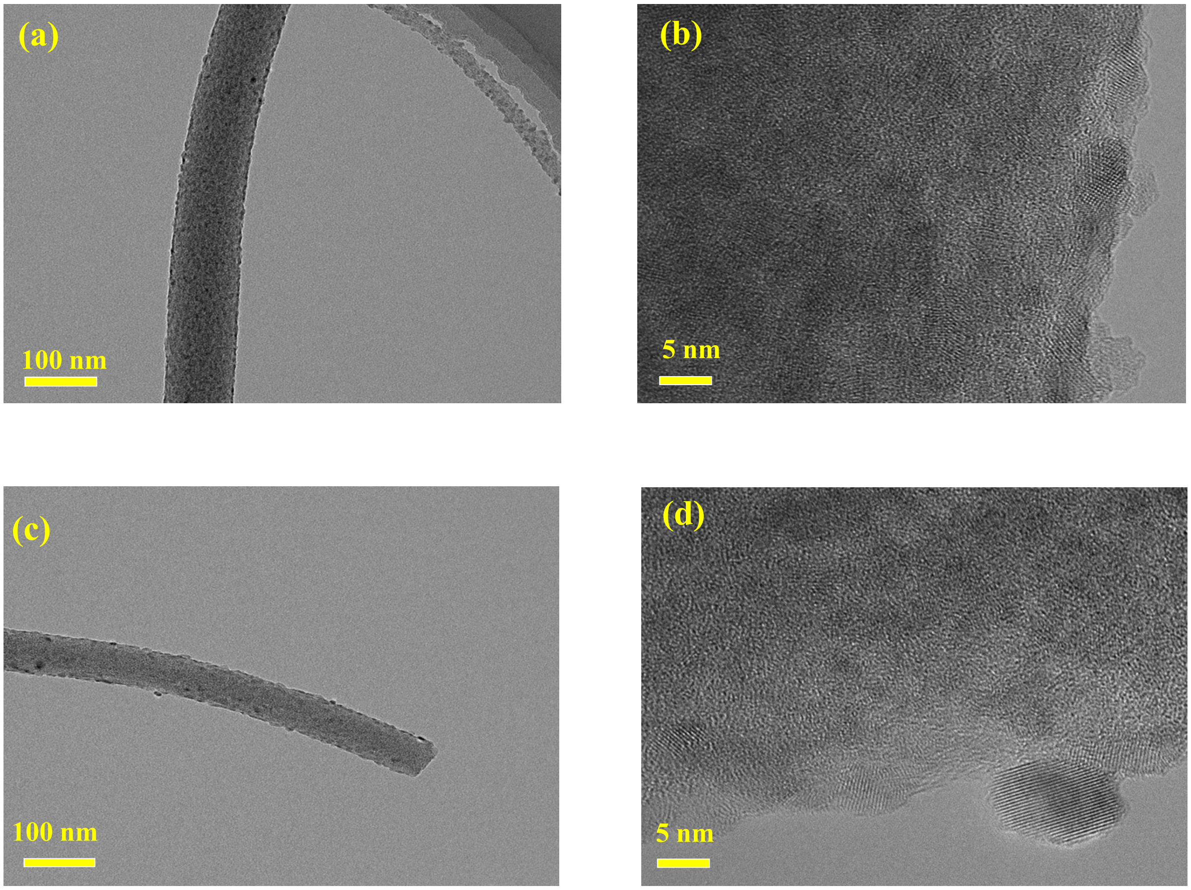

Fig. S10. TEM images of TiO_2/C NFs and Sb-TiO_2 /C NFs

Fig. S11. SEM images After long-term cycling

Table S1. Analysis of the content of Sb and Ti in Sb-TiO_2/C NFs by ICP-OES

**Sb-TiO_2/C NFs**	**Sample1**	**Sample2**	**Average content W(%)**
**Sb**	**7.59%**	**7.69%**	**7.64 wt%**
**Ti**	**25.71%**	**25.85%**	**25.78 wt%**
